# Prevention of Invasive Aspergillus Fungal Infections with the Suspension and Delayed-Release Tablet Formulations of Posaconazole in Patients with Haematologic Malignancies

**DOI:** 10.1038/s41598-018-20136-3

**Published:** 2018-01-26

**Authors:** Elisabeth Leclerc, David Combarel, Madalina Uzunov, Véronique Leblond, Christian Funck-Brentano, Noël Zahr

**Affiliations:** 1 Department of Pharmacology and CIC-1421, AP-HP, Pitié-Salpêtrière Hospital, F-75013 Paris, France; 2 Department of Haematology, AP-HP, Pitié-Salpêtrière Hospital, F-75013 Paris, France; 3 Department of Haematology, Sorbonne Université, UPMC Univ Paris 06, Faculty of Medicine, F-75013 Paris, France; 4 INSERM, CIC-1421 and UMR ICAN 1166, F-75013 Paris, France; 5 Department of Pharmacology and UMR ICAN 1166, Sorbonne Université, UPMC Univ Paris 06, Faculty of Medicine, F-75013 Paris, France

## Abstract

Posaconazole is a triazole antifungal used to prevent invasive fungal infections (IFIs) in patients receiving chemotherapy or haemotopoietic stem cell transplantation. Due to highly variable bioavailability of the oral suspension formulation, a delayed-release tablet was developed which showed improved bioavailability. A minimal target posaconazole plasma concentration of 0.7 mg/L is recommended for prophylaxis of IFIs. However, the relationship between plasma concentration of posaconazole and its efficacy against IFIs remains unclear. We analysed trough posaconazole concentrations and response against IFIs in 50 and 104 patients with haematologic malignancies receiving prophylactic posaconazole as the tablet or suspension formulation, respectively. Mean plasma concentration of posaconazole was 1.91 ± 1.06 mg/L and 0.82 ± 0.57 mg/L in the tablet and the oral suspension group, respectively (*p* < 0.0001). The percentage of patients reaching the minimal target concentration of 0.7 mg/L was 92.0% and 47.1% in the tablet and oral suspension groups, respectively (*p* < 0.0001). Emergent aspergillosis occurred in 9 (8.7%) patients in the suspension group and in none of the patients taking the tablet formulation (*p* = 0.032). Our results show a relationship between plasma concentrations of posaconazole and its prophylactic efficacy in patients with haematologic malignancies. Target posaconazole concentrations are reached more efficiently with the tablet than with the suspension formulation.

## Introduction

Posaconazole (PCZ) is a triazole derivative antifungal indicated in the prophylaxis of invasive fungal infections (IFIs) in patients undergoing chemotherapy or haemotopoietic stem cell transplantation and as second-line curative treatment of IFIs. PCZ has a broad antifungal spectrum, is better tolerated than fluconazole and itraconazole in neutropenic patients and is non-inferior to itraconazole in those with graft-versus-host disease. This favourable profile makes PCZ a major component of the antifungal therapeutic arsenal^1^.

PCZ oral suspension has a variable, pH- and dose-dependent bioavailability. Factors such as proton pump inhibitors co-administration, diarrhoea or mycosis, significantly reduce the absorption of PCZ oral suspension^2,3^. In contrast, high-fat meals or acidic beverages, can significantly increase PCZ absorption^4^. Studies have reported that less than 50% of patients receiving PCZ oral suspension reach the threshold concentration of 0.7 mg/L, corresponding to the minimal recommended exposure^5–9^.

In 2014, a gastro-resistant tablet formulation of PCZ was approved by the Food and Drug Administration and by the European Medicines Agency and made available for therapeutic use. PCZ delayed-release, formulated with a pH-sensitive polymer, has an improved oral bioavailability compared to the oral suspension and is not subject to pH-related drug interactions^10^. This improved absorption profile of the tablet formulation, together with its prolonged terminal elimination half-life of 25 to 30 h, allows once daily administration whereas the oral suspension requires two to four daily administrations^11^.

Studies have shown that tablets provide higher residual concentrations compared to the suspension formulation without increasing PCZ toxicity^9,12–15^. Therapeutic drug monitoring is recommended to ensure that patients reach sufficient PCZ exposure. Although it is recognized that recommendations are based on relatively weak evidence, prophylactic and curative recommended thresholds for PCZ plasma concentration are 0.7 mg/L and > 1 mg/L, respectively^16^. Whether the improved bioavailability of the tablet formulation is associated with a better prophylactic efficacy against IFIs, however, remains debated^9,15,17^.

The aim of this study was to assess whether the higher bioavailability of PCZ tablet formulation, compared to the suspension formulation, is associated with an increased proportion of patients reaching the target posaconazole prophylactic concentrations ≥ 0.7 mg/L and with an improved prevention of IFIs.

## Results

Data from 512 assays performed in 154 patients (60% males), aged 53.7 ± 13.5 years were available for analysis. PCZ was administered as the suspension in 104 patients and as the tablet formulation in 50. Ten patients were switched from suspension to tablets during their medical care. These groups were comparable in terms of age, clinical and biological characteristics (Table 1). PCZ was mainly administered due to haematological malignancies and most patients had undergone allogenic bone marrow transplantation. Mean (±sd) PCZ dose was 627 ± 143 mg per day in patients receiving the suspension and 290 ± 45 mg per day in patients receiving tablets.

**Table 1:** Demographic, clinical and biological characteristics of the studied population.

Variable	Tablet (n = 50)	Oral suspension (n = 104)
Age (yr) (mean ± sd)	54.9 ± 13.8	53.1 ± 13.3
Hematologic malignancy – Total (n)	50	100
Acute Myeloid Leukemia (n) (%)	5 (10.0)	18 (18.6)
Allograft (n) (%)	45 (90.0)	82 (82.0)
Severe chronic neutropenia	0	4
AST (mean ± sd)	35.0 ± 26.2	33.5 ± 16.7
ALT (mean ± sd)	46.4 ± 44.5	47.1 ± 33.7
Creatinine (µmol/L) (mean ± sd)	97.1 ± 42.3	83.3 ± 51.7
Diarrhoea (n) (%)	6 (12)	17 (16.3)
Tacrolimus co-prescription (n) (%)	4 (8)	3 (2.9)
Cyclosporine co-prescription (n) (%)	31 (62)	59 (56.7)

AST: aspartate aminotransferase; ALT: alanine aminotransferase.

Mean residual concentrations obtained with tablets was significantly higher than those obtained in patients receiving the oral suspension (1.91 ± 1.06 mg/L *vs*. 0.82 ± 0.57 mg/L; *p* < 0.0001**)**. The minimal target concentration of 0.7 mg/L was reached in 92.0% and 47.1% of patients in the tablet and oral suspension groups, respectively (*p* < 0.0001). In patients receiving the tablet formulation, those with diarrhoea (*n* = 6) had significantly lower plasma PCZ concentrations than those without diarrhoea (*n* = 44) 0.99 ± 0.49 mg/L *vs*. 1.98 ± 1.0 mg/L (*p* = 0.009).

**Figure 1:**
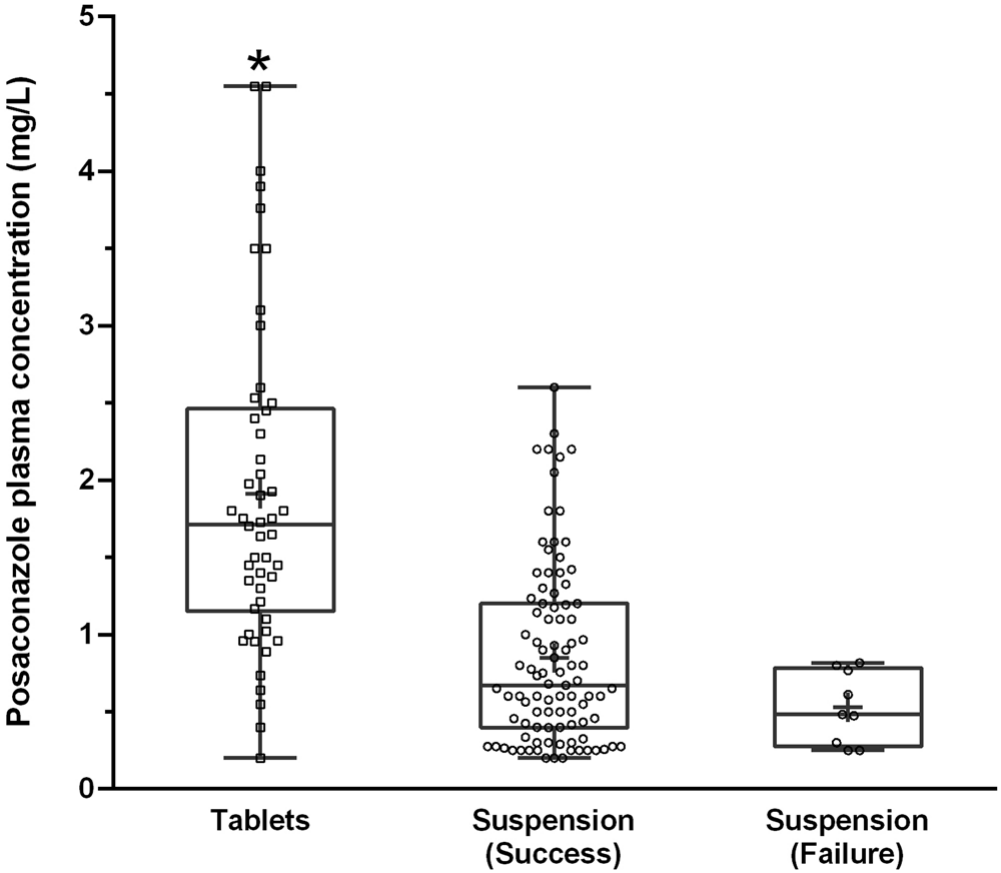
Posaconazole plasma concentrations in patients treated with the tablet formulation (*n* = 50) and with the suspension formulation (*n* = 104). Individual concentrations are shown together with the interquartile and extreme range, the mean (+) and median (horizontal line) values for each group. Patients receiving the suspension formulation are separated into those who experienced a treatment failure (*n* = 9) and those who did not (*n* = 95). *p < 0.0001 *vs*. other groups.

**Figure 2:**
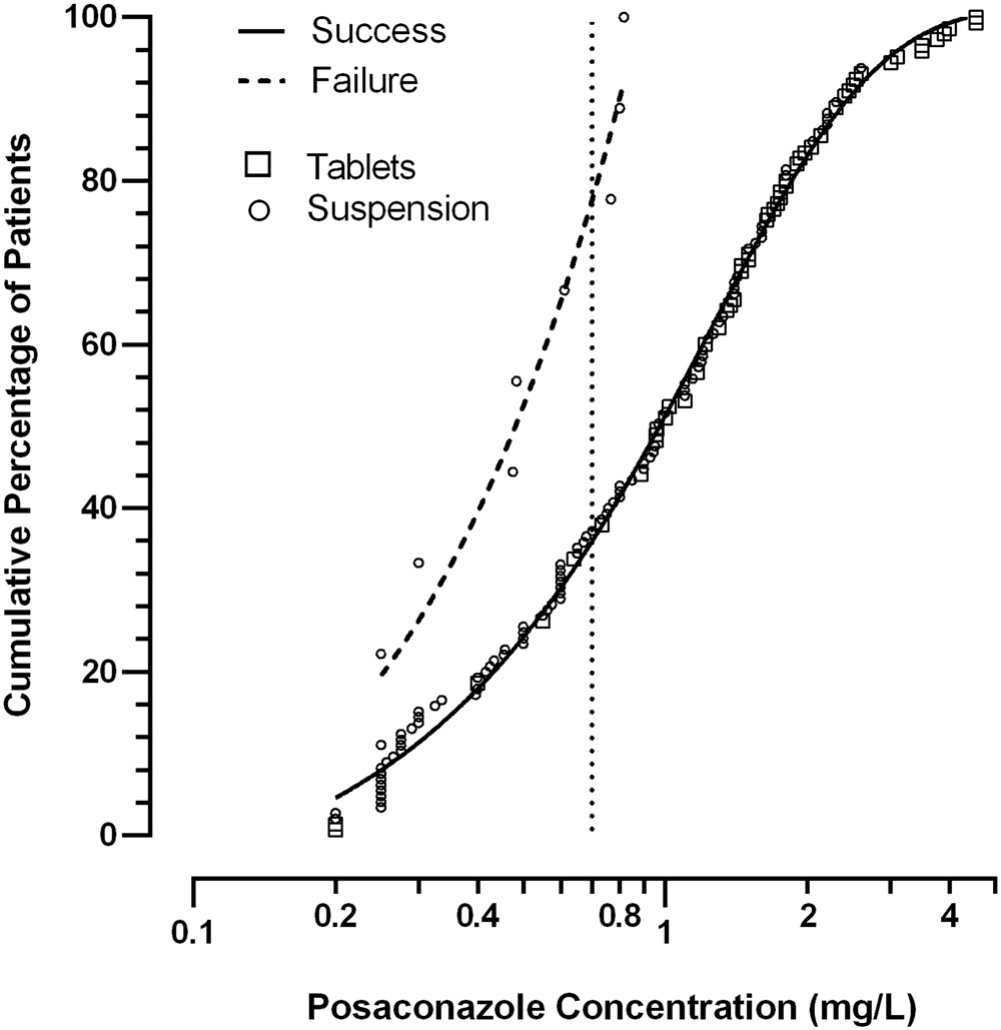
Cumulative percentage of patients with success (*n* = 145) and failure (*n* = 9) of prophylactic posaconazole treatment as a function of posaconazole plasma concentration. The dotted vertical line is placed at the recommended threshold of 0.7 mg/L for prophylactic treatment.

An invasive fungal infection (1 proven, 3 probable and 5 possible aspergillosis) occurred in 9 of 104 patients (8.7%; 95% confidence interval: 4.3–16.2%) in the oral suspension group and in none of the 50 patients receiving tablets, (*p* = 0.032). Figure 1 shows PCZ concentrations in patients in whom infection was prevented and in those who experienced an emergent infection. The mean residual concentration of PCZ was 0.53 ± 0.23 mg/L *vs*. 1.22 ± 0.93 in patients with and without IFIs, respectively (*p* < 0.03). Six of the 9 patients (66.6%) with an emergent infection and 53 of 145 patients (36.6%) without such an event did not reach the prophylactic target PCZ concentrations ≥ 0.7 mg/L (*p* = 0.087). Figure 2 shows the cumulative distribution of patients as a function of increasing PCZ concentration in responders and non-responders to IFIs prophylaxis.

**Figure 3:**
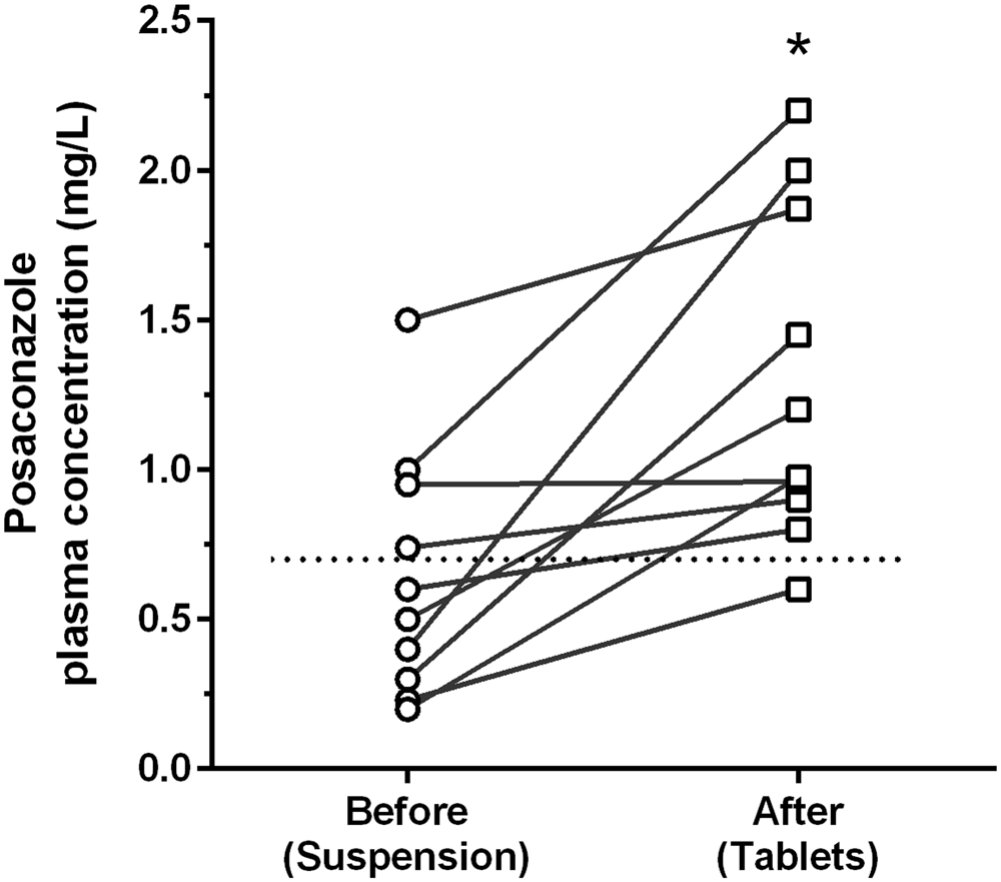
Mean posaconazole concentrations in the 10 patients who were switched from the suspension to the tablet formulation. The horizontal dotted line is placed at the recommended threshold of 0.7 mg/L for prophylactic treatment. *p = 0.002.

An increase of residual PCZ concentrations from 0.64 ± 0.41 mg/L to 1.30 ± 0.56 mg/L (*p* = 0.002) was observed in all 10 patients who were switched from oral suspension to tablets (Fig. 3). The target concentration threshold was reached in 4 and 9 of these 10 patients before and after the switch, respectively.

Increased exposure of posaconazole with the tablet formulation was not associated with hepatotoxicity (3 cases in 104 patients receiving the suspension and 1 case in 50 patients receiving tablets).

## Discussion

Our study shows for the first time that, compared to the suspension formulation, the higher residual plasma concentrations provided by the tablet formulation during prophylactic treatment with PCZ are associated with a reduction in emergent aspergillus infections. Dolton *et al*. previously showed a relation between PCZ concentration and therapeutic response in 72 patients treated with the suspension formulation^17^. Fungal infections were due to Candida or to an unknown pathogen. Fourteen patients also received posaconazole treatment for fungal infections caused by species from nine genera, including *Aspergillus fumigatus* (mixed infection with zygomycete). Median posaconazole concentrations in patients with zygomycete and *Aspergillus fumigatus* infection who failed treatment with posaconazole was 0.139 mg/L. Belling *et al*. did not find a statistically significant difference in therapeutic response between the suspension and the tablet formulation in 118 and 64 patients with haematological malignancies, respectively, who received prophylactic PCZ^15^. Cumpston *et al*. also did not find significant differences in therapeutic response in 118 and 32 patients treated with the suspension and the tablet, respectively^9^. In these previous studies, infections were not due to aspergillosis. It is therefore conceivable that our results may apply to the prophylaxis of Aspergillus infections and not to other pathogens.

In our study, the higher PCZ exposure with the tablet formulation compared to the suspension formulation presumably contributed to the reduced number of treatment failures with tablets. However, among the 9 treatment failures, 3 patients taking the oral suspension reached the threshold concentration (≥0.7 mg/L) but nonetheless developed an infection (Fig. 2). Their PCZ concentrations were 0.77, 0.80 and 0.82 mg/L. This supports the recently expressed view^11^ that the recommended prophylactic threshold concentration of 0.7 mg/L may be too low.

The study also confirms that, compared to the standard oral suspension formulation of PCZ, administration of the delayed-release tablet formulation is associated with an increase in PCZ oral bioavailability leading to an improved exposure of patients above therapeutic thresholds during prophylactic therapy but does not increase the hepatotoxicity of PCZ^9,18,19^. Our results are consistent with those of Jung *et al*.^20^ who found that switching from oral suspension to tablets increases the proportion of patients reaching target prophylactic exposure without treatment-limiting hepatotoxicity related to increased plasma levels.

Therapeutic drug monitoring is justified in patients treated by PCZ oral suspension due to its low bioavailability to ensure an optimised PCZ exposure. Although bioavailability is improved with the tablet formulation, 8% of patients receiving the tablet formulation in our study remained under the minimal recommended threshold of exposure. Also, despite higher concentrations in the tablet than in the suspension group, high inter-individual variability of PCZ concentrations persisted with tablets (Figs 1, 2 and 3). This indicates that it is still appropriate to perform therapeutic drug monitoring in patients treated with PCZ tablets.

In conclusion, the present study shows that the tablet formulation of PCZ provides better prevention of Aspergillus fungal infections than the suspension formulation in patients with haematologic malignancies. Despite a limited number of cases with treatment failure in our study, it appears that the recommended prophylactic threshold of 0.7 mg/L for plasma concentrations of posaconazole may be too low.

## Methods

We conducted an observational, single-centre study on all patients with haematologic malignancies who were treated with PCZ for antifungal prophylaxis from January 2013 to April 2017. They received 600 to 800 mg of PCZ per day as the oral suspension, or 100 mg to 300 mg once a day of PCZ as the gastro-resistant tablets formulation. Patients’ medical records were retrospectively reviewed for demographic, clinical and biological characteristics. PCZ plasma concentrations were measured using residual concentrations after at least 7 days of treatment, i.e. under steady-state condition^16^. When several concentrations were available in a given patient, the mean of these concentrations was used in the analyses. Treatment failure was defined as a possible, probable or proven infection according to EORTC criteria^21^. Hepatotoxicity was defined as an increase in alanine transaminase (ALT) to 3 times the upper limit of normal and a 30% increase in the baseline value in association with PCZ prophylaxis.

A validated high-performance liquid chromatography-tandem mass spectrometry assay was used to measure PCZ plasma concentrations^22^. Calibration curves were linear over the concentration range of 0.25 to 5.05 mg/L. Precision and accuracy errors tested at 0.64 and 3.33 mg/L were below 15%.

Statistical analyses were performed using XLSTAT (Addinsoft, New-York, NY, USA). A t-test was used to compare PCZ concentrations in patients receiving the suspension or the tablet formulation. The Mann-Whitney test was used to compare PCZ concentrations in the failure and the success group. The sign-test was used to compare PCZ concentrations in patients switched from the suspension to the tablet formulation. Fisher’s exact test was used to compare the proportion of patients reaching various binary targets. A *p* value > 0.05 was considered significant.

The study was performed in accordance with French regulations on non-interventional observational studies which do not require patient’s consent when analysing data obtained from routine care and approval was obtained from the Commission Nationale de l’Informatique et des Libertés (n°1491960v0).
